# Ethyl Pyruvate Ameliorates The Damage Induced by Cyclophosphamide on Adult Mice Testes

**DOI:** 10.22074/ijfs.2016.4772

**Published:** 2016-04-05

**Authors:** Zahra Bakhtiary, Rasoul Shahrooz, Abbas Ahmadi, Farhad Soltanalinejad

**Affiliations:** Department of Basic Sciences, Faculty of Veterinary Medicine, Urmia University, Urmia, Iran

**Keywords:** Testis, Cyclophosphamide, Ethyl Pyruvate

## Abstract

**Background:**

Cyclophosphamide (CP) is a chemotherapy drug which causes deleterious effects on testicular tissue and increases free radicals in the body. The aim of this study is to investigate the protective effects of ethyl pyruvate (EP) on testicular improvement in CP treated animals.

**Materials and Methods:**

In this experimental study, 15 male mice (6-8 weeks) were divided into 3 groups. The control group received normal saline (0.1 ml/day), intraperitoneal (IP), CP group received CP (15 mg/kg/week, IP), and the CP+EP group received EP (40 mg/kg/day, IP) plus CP. After 35 days, we assessed serum total antioxidant capacity (TAC) along with histomorphometric and histochemical analyses of the testicles.

**Results:**

The mean thickness of the germinal epithelium, diameter of seminiferous tubules, and the number of Leydig cells in the CP+EP group were higher than those of the CP group (P<0.05). The number of the mast cells in the CP+EP group significantly reduced compared with the CP group (P<0.05). Alkaline phosphatase (ALP), periodic acid-schiff (PAS) positive reactions and lipid granules in cytoplasm of the Leydig cells in the CP group increased compared with the other groups (P<0.05). TAC in the CP group significantly reduced compared with the other groups (P<0.05).

**Conclusion:**

This study showed the ability of EP to reduce the destructive side effects of CP in the adult mice reproductive system.

## Introduction

A number of chemotherapeutic drugs such as cyclophosphamide (CP) that are used for neoplastic patients leave toxic side effects in various systems of the body, including the male reproductive system. Chemotherapy with CP disrupts reductive reactions in tissues and creates oxidative stress (1,3) as an alkylating agent, finally reducing fertilization in patients under treatment (4,5). CP is converted into its active metabolites with the action of oxidase enzymes in the liver (6). Phosphoramide mustard and acrolein are active metabolites of CP (7). All anticancer effects of CP related to phosphoramide mustard and its toxic effects are related to acrolein (8). Acrolein, as a toxic metabolite of CP, interferes with the antioxidant system of tissues (9), producing a high level of reactive oxygen species (ROS) (2,10). The cytotoxic effects of CP particularly target rapidly proliferating cells; hence the testicles are a target for the destructive effects of this drug (11). According to the importance of reproduction in humans and the use of antioxidants to decrease or eliminate free radicals produced by CP, we have chosen ethyl pyruvate (EP), a synthetic antioxidant with different therapeutic properties, for this study. EP is a primary antiinflammatory, anti-oxidant molecule which improves local inflammation in the liver and, as a result, reduces secondary hepatic injury caused by acute pancreatitis (12). In addition, EP has a protective effect on nerves against paraquat toxicity (13). The effects of EP on oxidative stress caused by CP have not been studied in testicular tissue. Hence, this study evaluated the protective effects of EP on improvement of the testicles and serum antioxidants in CP treated animals. 

## Materials and Methods

### Drugs and chemicals

CP (500 mg) was obtained from Baxter, Germany. EP was purchased from Sigma Aldrich (MO, USA). 

### Animals

In this experimental study, 15 adult male mice Naval Medical Research Institute (NMRI) mice (6-8 weeks) that weighed 20-25 g were used. The animals were randomly divided into three groups and maintained under standard conditions at 22 ± 2°C, 30-60% humidity, with 14 hours daylight and 10 hours darkness. All performed experiments in this study were in accordance with the guidance of the Ethical Committee for Research on Laboratory Animals at Urmia University. 

### Experimental design

Animals were divided into three groups, as follows: i. Control group (C) received normal saline [0.2 ml/day, intraperitoneal (IP)], ii. CP group received (15 mg/kg/week, IP) of CP, and iii. CP+EP group received EP (40 mg/kg/day, IP) plus CP (15 mg/kg/week, IP). 

After 35 days, all mice were anesthetized and euthanized with ketamine (25 mg/kg, IP) after which serum and testicular samples were taken for further analyses. 

### Biochemical analysis

After the serum samples centrifuged at 3000 for 5 minutes twice, total antioxidant capacity (TAC) measured according to the Benzian method (14). 

### Histological analyses

The right testicles were fixed in 10% formal saline for 72 hours, after which the samples were dehydrated, cleared, and embedded in paraffin. Paraffin sections were prepared (6-7 µm in thickness) and stained with hematoxylin and eosin (H&E) for histomorphometry analyses with an Olympus light microscope (BH-2 model) and calibrated, graded objective lens. We measured the germinal epithelium thickness, diameter of the seminiferous tubules, and the number of Leydig cells in 1 mm ^2byusing^a latticed objective lens. We investigated the interstitial tissue in terms of edema and hyperemia, and seminiferous tubules in terms of morphological features such as the germinal epithelium. Toluidine blue staining was used to assess the mean number of mast cells (15). 

### Histochemical analyses

Oil red-O staining was performed on formalin buffer fixed specimens and frozen sections to evaluate the rate of lipid foci (brilliant red) supplement in Leydig cells and germinal epithelium (15). Other sections were stained with alkaline phosphatase (ALP) (16). ALP staining of testis tissue causes a dark brownish color reaction. Granules that contain carbohydrate compounds were stained with periodic acid-schiff (PAS) (17). PAS positive granules stained a brilliant red color. 

### Statistical analysis

The data were analyzed by SPSS software (version 20, SPSS Inc., USA); one-way ANOVA and the Bonferroni test were used. A P<0.05 was considered significant. 

## Results

### Ethyl pyruvate ameliorates the germinal epithelium disarrangement induced by cyclophosphamide in the EP+CP group 

**Fig.1:**
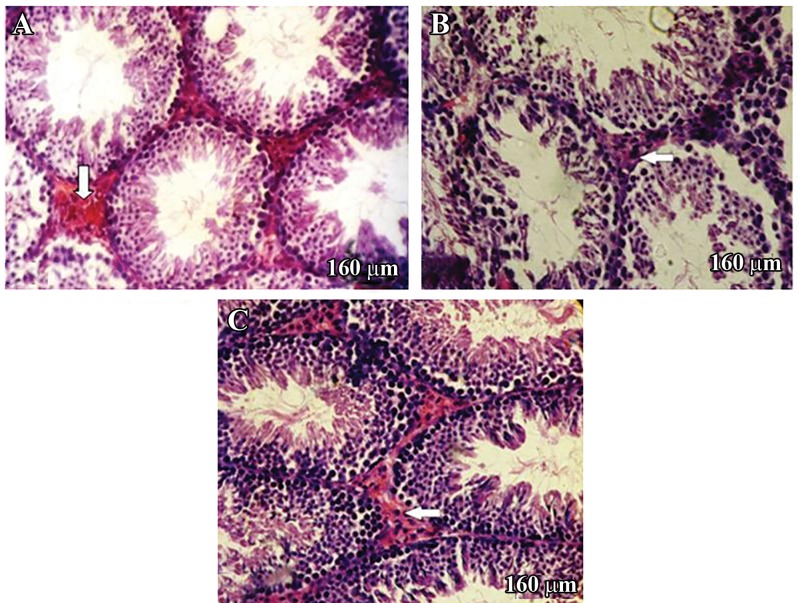
Histological changes in the: A. Control (C) group, B. Cyclophosphamide (CP) and C. CP+ethyl pyruvate (EP) groups. Leydig cells present in interstitial tissue (thick arrows), which was prominent in the C and CP+EP groups. The cytoplasm stained intensely with eosin (A) compared with the CP (B). Notice the germinal epithelium that is integrated in the CP+EP group (C), whereas it was disorganized in the CP group (B) (H&E; ×400).

Histological studies showed the presence of edema in the interstitial tissue, disruption of spermatogenic cells, and reduction of germinal epithelium height in most seminiferous tubules in the CP group compared to the control group. These conditions clearly improved in the CP+EP group (Fig .1). 

### Ethyl pyruvate ameliorates the thickness of germinal epithelium and seminiferous tubules in the EP+cyclophosphamide group

**Fig.2:**
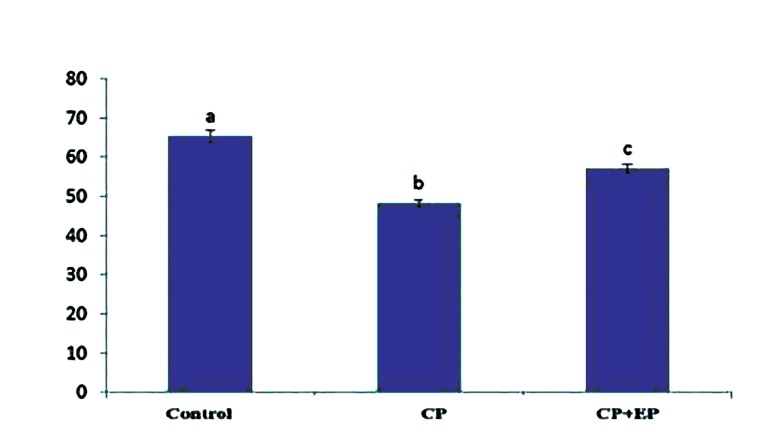
Germinal epithelium thicknessin testis (mean ± SE, μm). Non-similar letters (a, b, c) indicate significant differences (P<0.05). CP; Cyclophosphamide and EP; Ethyl pyruvate.

**Fig.3:**
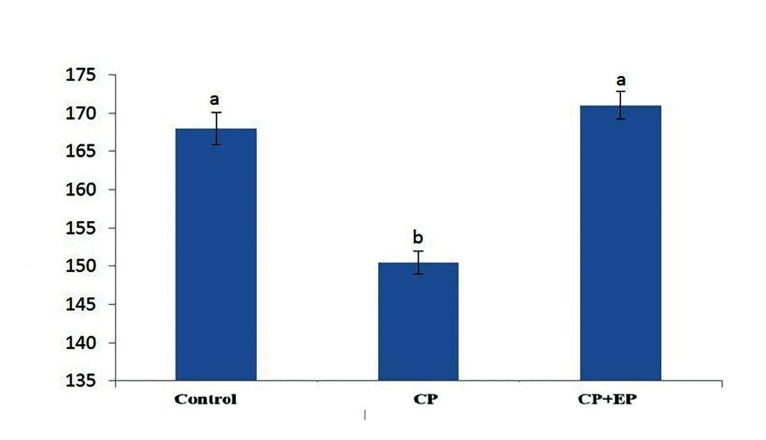
Diameter of seminiferous tubules (mean ± SE, μm). Nonsimilar letters (a, b, c) indicate significant difference (P<0.05). CP; Cyclophosphamide and EP; Ethyl pyruvate.

Morphometric studies showed that the germinal epithelium in the CP group was disarranged and disrupted. Its thickness significantly reduced compared to the control and EP+CP groups (P<0.05, Fig .2). There were significantly decreased seminiferous tubule diameters in the CP group compared with the other groups (P<0.05). However the control and EP+CP groups did not significantly differ (Fig .3).

### Ethyl pyruvate increased the number of Leydig cells in the cyclophosphamide+EP group

**Fig.4:**
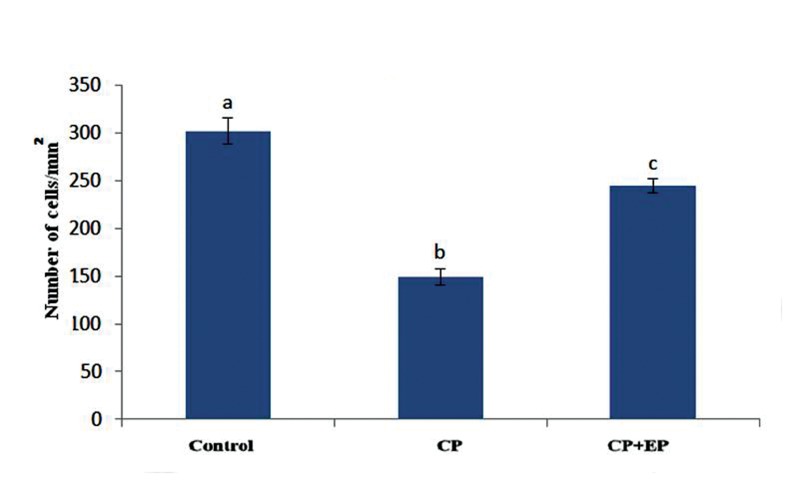
Number of Leydig cells in testis (mean ± SE). Non-similar letters (a, b, c) indicate significant difference (P<0.05). CP; Cyclophosphamide and EP; Ethyl pyruvate.

This study showed that Leydig cells were in the interstitial tissue, almost accumulating around the blood vessels. They had an extensive acidophilic cytoplasm visualized by H&E staining, with spherical, euchromatic nuclei in the middle of the cells (Fig .1). There were a significantly reduced mean number of Leydig cells in the CP group compared with the other groups (P<0.05, Fig .4).

### Ethyl pyruvate ameliorates the histochemical feature of the testis in cyclophosphamide treated mice

**Fig.5:**
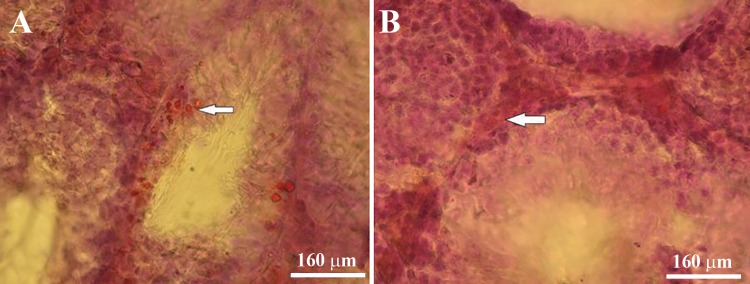
Lipid accumulation shown as red granules in the cyclophosphamide (CP), CP+ethyl pyruvate (EP) groups is detected in the A. Cytoplasm of spermatogenic and Sertoli cells in testes of the CP group (arrow) and B. Cytoplasm of Leydig or interstitial endocrine cells (arrow) in the CP+EP group (Oil red-O staining, ×400).

**Fig.6:**
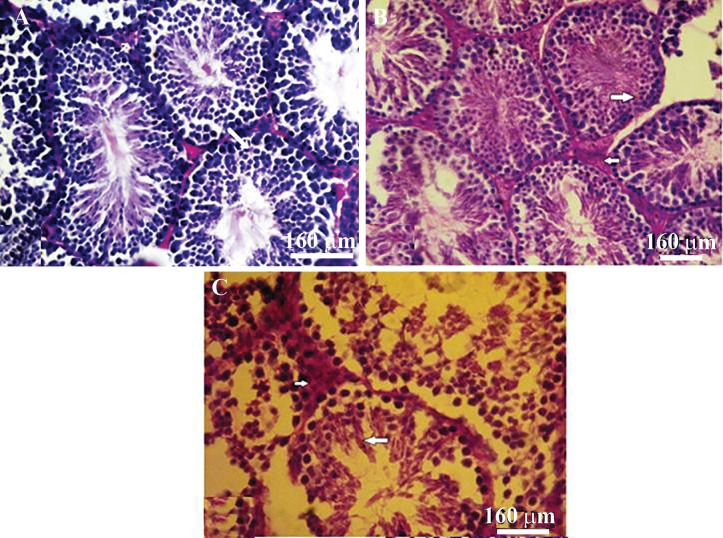
Periodic acid-schiff (PAS) reaction in the A. Control (C) group, B. Cyclophosphamide (CP) and C. CP+ethyl pyruvate (EP) groups. B. Accumulation of carbohydrate as red granules in the cytoplasm of Leydig cells (small arrows) shown in the CP group. PAS reaction was faintly observed in spermiogenic cells of the B. CP group (thick arrow) and in the cytoplasm of spermatogenic cells in the control, CP and CP+EP groups (thick arrows) (magnification: ×400).

**Fig.7:**
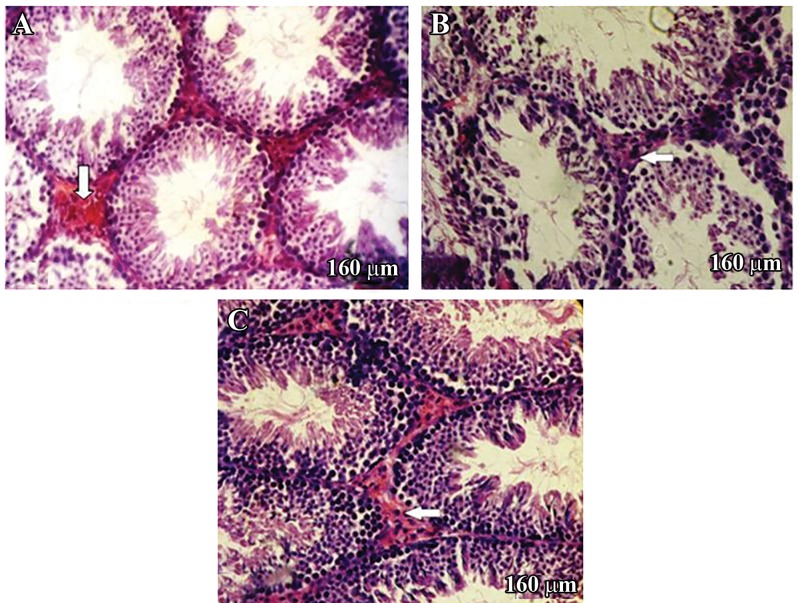
Alkaline phosphatase (ALP) reaction in the cyclophosphamide (CP) and CP+ethyl pyruvate (EP) groups shown as dark granules in Leydig cells located in the interstitial tissue of mice testis in the A. CP group (arrow), while this reaction was scant in the B. CP+EP group (magnification: ×400).

According to oil red-O staining, lipid granules in the cytoplasm of Leydig cells in the CP group increased compared to the other groups. Accumulation of lipid in the cytoplasm of spermatogenic cells adjacent to the basal lamina of the seminiferous tubules was observed in the CP group (Fig .5). PAS staining showed an increased PAS positive reaction in cells adjacent to the lumen of seminiferous tubules and Leydig cells in the CP group compared with the control and CP+EP groups (Fig .6). Reaction of ALP as dark brown fine granules in the interstitial tissue of the testicle was observed in the CP group; this reaction considerably reduced in the CP+EP group (Fig .7).

### Ethyl pyruvate reduces the number of mast cells during oxidative stress

**Fig.8:**
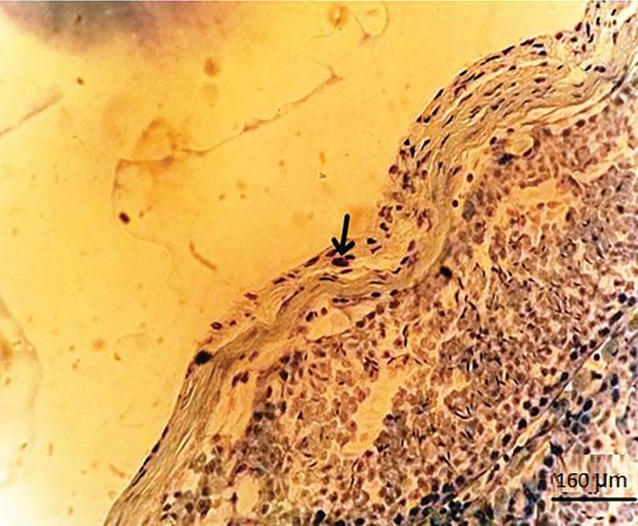
Mast cell localization in testicular capsule. Mast cell with dark purple granule that occupied the cytoplasm in the testicular capsule of the cyclophosphamide (CP) group (arrow). (Toluidine blue staining, magnification: ×400).

**Fig.9:**
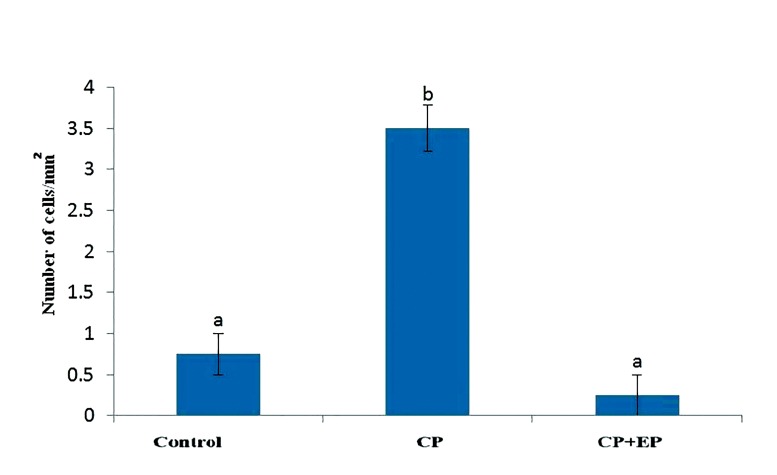
Number of mast cells in the testicular capsules (mean ± SE). Non-similar letters (a, b, c) indicate significant difference (P<0.05). CP; Cyclophosphamide and EP; Ethyl pyruvate.

The numbers of mast cells in the testicular capsule were determined by toluidine blue staining. We observed that the cytoplasm of the mast cells were full of dark reddish violet granules (metachromatic) in the testicular capsule (Fig .8). There was a significantly higher mean number of mast cells in the CP group compared to the control group (P<0.05), while EP in the CP+EP group reduced the mean number of these cells to a level comparable to the control group (P<0.05, Fig .9).

**Fig.10:**
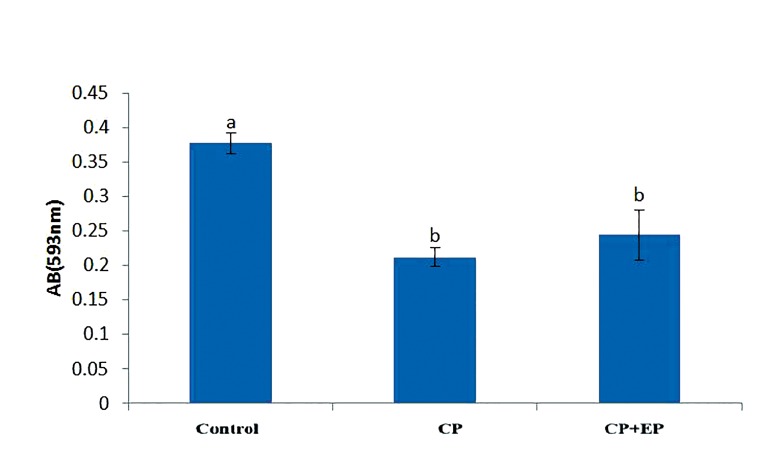
Total antioxidant capacity (TAC) levels in different groups (mean ± SE). Non-similar letters (a, b, c) indicate significant difference (P<0.05).

## Discussion

According to previous studies, the toxic side effects of CP cause histological, histochemical and serological changes (2, 18, 19). Chemotherapy causes long-term or permanent azoospermiadue to destruction and damage of the testicular germ cells (20). We have observed that damage to germinal cells with CP was a main reason for the reduction in diameter of seminiferous tubules and height of germinal epithelium in the CP group. EP, with its antioxidant effects, caused a significant increase in these two parameters in the CP+EP group.

The toxic effect of chemotherapy on Leydig cells and indirect effect of damaging spermatogenic cells on negative performance of Leydig cells (21) justified the significant reduction in numbers of these cells in the CP group compared to the other groups. Accumulation of neutral lipids in the cytoplasm of spermatogenic cells adjacent to the basal lamina of seminiferous tubules in the CP group compared with other groups could be related to destruction of spermatogenic cells and accumulation of unconsumed lipid for biosynthesis of steroid hormones (22). Accumulation of lipid in this region might be related to increased phagocytosis of the apoptotic spermatogenic cells by Sertoli cells (23).

On the other hand, it has been shown that oxidative mechanisms increase active species of oxygen and lipid peroxidation by inactivating microsomal enzymes (24). The role of CP in production of free radicals and reduction of antioxidants (19, 25) was the logical reason for reduction of serum TAC in the CP group compared to the other groups.

Allergic and immunologic stimulations caused by prescription of CP increases the mean number of mast cells in the testicle capsule and consequently increase production of free radicals with degranulated mast cells, leading to reproductive disorders (26). On the other hand, degranulation of mast cells following acute physical and chemical stresses lead to secretion of histamine which increases permeability of the blood vessels (27). Increases in permeability of blood vessels and tissue edema by stimulation of apoptosis in endothelial cells (28, 29) and smooth muscular cells of the blood vessel wall (30, 31) also occur. With respect to results of the above studies, we have observed an increased number of mast cells in the testicle capsule, edema, and hyperemia in the interstitial tissue of the CP group. These would be expected side effects of CP on the testicles, which considerably reduced in the CP+EP group.

ALP enzyme activity in the testicles of rats with varicocele increased with degeneration of the reproductive cells (32). Therefore, the increased ALP reaction observed in the CP group was affected by the destructive effects of CP on reproductive cells of the testicle. Reduction of this reaction in the CP+EP group has supported results of previous studies. These degenerative changes in testicular tissue reduce glucose transmitters (33). CP disrupts transmission of glucose to the seminiferous tubules and spermatogenic cells, which have high a mitotic activity and a negative reaction against PAS staining due to the damage of these transmitters.

## Conclusion

This study showed the protective effects of EP in the testis of CP treated mice.
